# Imaging Studies of Aging, Neurodegenerative Disease, and Alcoholism

**Published:** 1995

**Authors:** Jamie L. Eberling, William J. Jagust

**Affiliations:** Jamie L. Eberling, Ph.D., is a staff scientist at the Center for Functional Imaging, Lawrence Berkeley National Laboratory, Berkeley, California. William J. Jagust, M.D., is head of the Neuroscience Group at the Center for Functional Imaging, Lawrence Berkeley National Laboratory, Berkeley, and a professor in the Department of Neurology, University of California, Davis, California

**Keywords:** neuroimaging, aging, dementia, brain atrophy, CNS degenerative disorder, Alzheimer’s disease, computed x-ray tomography, magnetic resonance imaging, positron emission tomography, single photon emission computed tomography, Parkinson’s disease, Korsakoff’s syndrome

## Abstract

Neurodegenerative diseases such as Alzheimer’s disease, disorders such as alcoholism, and the aging process can lead to impaired cognitive function and dementia. Researchers and clinicians have used noninvasive imaging techniques to determine the structural and physiological alterations in the brain that are associated with these conditions. Analyses of the brain’s structure have found that shrinkage (atrophy) of the brain tissue is characteristic for all conditions associated with dementia, but that the specific locations of atrophied brain structures vary among different neurodegenerative diseases and alcohol-induced disorders. Similarly, studies analyzing the metabolism in various brain structures have found that, depending on whether dementia was induced by neurodegenerative diseases, alcoholism, or aging, the affected brain structures vary slightly. Based on such studies, researchers and clinicians now can more accurately define different types of dementia and predict their clinical course.

Neurodegenerative diseases such as Alzheimer’s disease (AD) (see sidebar, p. 280), disorders such as alcoholism, and even the normal aging process can severely impair a person’s cognitive functioning. These cognitive disturbances frequently are accompanied by structural and physiological changes in the brain. Considerable advances in brain imaging technology have provided researchers and clinicians with new tools to study these changes and relate them to cognitive disturbances. Among the most commonly used techniques are computed tomography (CT) and magnetic resonance imaging (MRI), which allow evaluation of the brain structure. Single-photon emission computed tomography (SPECT) and positron emission tomography (PET) allow researchers to visualize brain functioning (e.g., the rate of metabolism). (For more information on these techniques, see the article by Doria, pp. 261–265.)

**Dementia and Its Causes**

The term “dementia” denotes any condition characterized by severe global intellectual impairment, including loss of memory functions and abstract thinking, personality changes, disruption of social skills, and other impairments of higher brain functions. Dementia can have a variety of causes, including alcoholism and other drug abuse, neurodegenerative diseases, and brain injuries or tumors.

***Alcoholism***

Alcoholism can lead to several degrees of cognitive dysfunction and pathological changes in the brain structure. Most commonly, chronic alcohol abuse is associated with relatively subtle cognitive and motor deficits (Martin et al. 1986). More rarely, alcoholism leads to alcoholic dementia, a neurobehavioral syndrome marked by a global loss of intelligence. In contrast, alcoholics with Korsakoff’s syndrome (KS) exhibit a more specific memory dysfunction marked by a permanent inability to remember new facts and events for more than a few seconds (i.e., anterograde amnesia) as well as deficits in nonmemory realms, such as temporal discrimination, spatial organization, abstraction, and initiative (Squire 1982). In addition to the severe deficits that define alcoholic dementia and KS, both conditions include the milder dysfunctions associated with chronic alcoholism.

***Neurodegenerative Diseases***

Neurodegenerative diseases (i.e., diseases in which parts of the nervous system are progressively destroyed) potentially resulting in dementia include Alzheimer’s disease (AD), Huntington’s disease (HD), and Parkinson’s disease (PD). AD, which occurs mainly in people over age 65 and is the most common cause of dementia, is characterized by progressive cognitive and intellectual deterioration (e.g., confusion, memory failure, and disorientation). These symptoms are accompanied by pathological changes in the brain, such as the degeneration of nerve fibers in certain brain regions (i.e., neurofibrillary tangles) and the formation of protein aggregates (i.e., senile plaques) in the brain tissue. HD is an inherited disease that usually develops during middle age. It is characterized by mental deterioration and involuntary, purposeless, rapid movements (e.g., flexing and extending the fingers or grimacing). PD, which primarily develops in people over age 60, is characterized mainly by rapid shaking of certain muscles (i.e., tremors) and a shuffling gait. Not all PD patients, however, show mental deterioration. Pathological changes in the brains of PD patients include the destruction of nerve cells in certain brain regions (e.g., the basal ganglia) and depletion of the neurotransmitter dopamine in brain structures that normally contain high dopamine levels (e.g., the striatum).

***Brain Injury***

After AD, ischemic infarcts in the brain, also called ischemic strokes, are the most common cause of dementia. The term “stroke” applies to all conditions during which the blood supply to a brain region is interrupted, leading to the death of surrounding brain tissue. During an ischemic infarct the blood flow is interrupted by a block in one of the blood vessels. The resulting condition of cognitive deterioration is called ischemic vascular dementia. The second type of stroke is the hemorrhagic stroke, which is caused by bleeding within the brain due to a ruptured blood vessel.

—Jamie L. Eberling and William J. Jagust

ReferencesMartinPRAdinoffBWeingartnerHMukherjeeABEckardtMJAlcoholic organic brain disease: Nosology and pathophysiologic mechanismsProgress in Neuro-Psychopharmacology and Biological Psychiatry101471641986287549010.1016/0278-5846(86)90069-2SquireLRComparisons between forms of amnesia: Some deficits are unique to Korsakoff’s syndromeJournal of Experimental Psychology: Learning, Memory, and Cognition8560571198210.1037//0278-7393.8.6.5606218221

Brain imaging techniques have both clinical and research applications. Clinicians use imaging technology as a diagnostic tool to distinguish between different types of dementia^1^ (e.g., dementia due to AD or alcoholism), allowing them to more accurately determine the patient’s prognosis, prescribe appropriate therapy, and provide adequate counseling. Researchers, on the other hand, can use brain imaging to investigate pathological metabolic changes associated with natural aging processes and with age-related or neurodegenerative diseases. This article summarizes some of the findings of such analyses, which have focused mainly on the effects of aging and on dementia caused by neurodegenerative diseases. Comparatively less research has analyzed how chronic alcohol use affects brain structure and physiology and how these effects correlate with cognitive deficits observed in alcoholics. However, studies of the effects of aging may be relevant to alcohol research because several researchers have postulated that alcoholism may accelerate normal aging processes or induce premature aging of the brain (For a review, see Evert and Oscar-Berman 1995).

## Structural Brain Analyses

### CT Studies of AD Patients

Both CT and MRI allow researchers to analyze brain structure and therefore can help detect abnormalities in the brain that may cause dementia, such as large lesions, damaged blood vessels, structural asymmetries, and abnormalities of the ventricles (i.e., cavities in the brain that are filled with cerebrospinal fluid [CSF]). CT particularly is useful in evaluating dementia resulting from large cortical infarcts (i.e., regions in which brain tissue dies because the blood supply is interrupted) but is less suitable for evaluating dementing diseases. For example, AD and ischemic vascular dementia (IVD) (see sidebar), in which the structural damage is more subtle, are not readily detectable using CT. Moreover, CT cannot identify some lesions because of its low contrast sensitivity (i.e., the signals of diseased and normal tissues are similar on the image). Nevertheless, CT often is used to rule out potentially reversible causes of dementia, such as tumors and other types of structural abnormalities that CT can detect easily.

Despite the technology’s limitations, CT studies of AD patients have demonstrated that the primary structural changes associated with the disease are shrinkage (i.e., atrophy) of the cerebral cortex and enlargement of the ventricles. The rate at which the volume of gray matter^2^ in the cortex decreases and the volume of CSF in the ventricles and in the fissures on the brain’s surface (i.e., sulci) increases determines the rate at which dementia progresses in AD patients (Luxenberg et al. 1987). Generalized cortical atrophy and ventricular enlargement, however, also occur in other types of dementia (e.g., IVD) as well as in normal aging; to distinguish AD from these conditions, physicians must analyze the extent of atrophy in specific regions of the cortex. For example, regional atrophy in the temporal lobe^3^ can accurately distinguish the effects of AD from those of normal aging (Kido et al. 1989). Alternatively, a physician could take several CT measurements over a period of time to document the rate of ventricular enlargement; more rapid changes in ventricle size indicate AD and distinguish it from slower, normal aging processes. Both of these types of CT measurements are fairly labor intensive and expensive, however, and therefore are not used routinely in the diagnosis of AD.

### CT Studies of Alcoholic Patients

To better understand the effects of chronic alcohol consumption on the brain, neuroimaging studies have attempted to relate structural brain alterations to clinical and cognitive changes associated with alcoholism, just as changes in the brains of AD patients have been related to those patients’ cognitive deficits. Several CT studies have demonstrated cortical atrophy and ventricular enlargement in alcoholic patients and reported relationships between structural changes and specific cognitive impairments. For example, Shimamura and colleagues (1988) reported atrophy of the frontal lobe and of an area near the Sylvian fissure (i.e., a fissure on the brain surface that separates the frontal lobe from the temporal lobe) both in alcoholics with Korsakoff’s syndrome (KS) (see sidebar) and in nondemented alcoholics. In addition, KS patients showed evidence of tissue loss in regions other than the cortex (i.e., subcortical regions), such as the thalamus, and of increased volume of the third ventricle, which is located in the center of the brain, near the thalamus. The degree of frontal lobe atrophy and tissue loss in the thalamus correlated with the KS patients’ performance on memory tests.

Alcohol’s effects on brain structure also may depend on the drinker’s age. When alcoholics were compared with age-matched nonalcoholics, increases in CSF volume—which reflect a loss of brain tissue—in the alcoholics appeared to be greater in older subjects than in younger subjects (Pfefferbaum et al. 1993; also see the article by Rosenbloom et al., pp. 266–272). These findings suggest that the aging brain may be increasingly vulnerable to the effects of alcohol. Other research has indicated that some of the alcohol-related structural changes may be partially reversible with abstinence (Carlen et al. 1978).

### MRI Studies of Aging and Dementia

MRI provides structural brain images that are superior to CT in several ways. For example, the contrast is greater in MRI images than in those produced using CT, allowing better distinction between gray and white matter, improved identification of white-matter lesions and subcortical infarcts, and more accurate volume determination of subcortical gray-matter structures (e.g., the thalamus). MRI also can distinguish better between adjacent structures (i.e., has better spatial resolution) than CT. Moreover, in contrast to CT, MRI generates three-dimensional data sets that can produce images of any structure from any angle.

Because of their excellent contrast, MRI images can, for example, detect changes in the white matter near the ventricles; these alterations often occur in AD and IVD patients (and, to a lesser extent, during normal aging) and may be associated with specific cognitive deficits. In addition, physicians can use MRI to distinguish IVD, which is characterized by small white-matter lesions that often are not visible on CT scans, from AD and thus improve the patient’s diagnosis and treatment. Although MRI is useful to researchers for identifying structural damage (e.g., white-matter changes) in patients with dementia, the clinical, diagnostic value of these observations often is limited and must be complemented by a thorough clinical evaluation. For example, the presence of white-matter lesions does not preclude a diagnosis of AD. In fact, using MRI, Harrel and colleagues (1987) observed white-matter lesions in 36 percent of AD patients studied. This finding could reflect either a coexistence of AD and IVD in many patients or the nonspecific nature of white matter changes.

The high spatial resolution of MRI images also allows researchers to determine the size (i.e., volume) of small brain structures (e.g., the hippocampus and the amygdala) that are known to be affected in AD patients. For example, hippocampal atrophy can distinguish AD patients from age-matched control subjects (Seab et al. 1988; Jack et al. 1992) and thus assist in the diagnosis of AD. Moreover, Golomb and colleagues (1993) used MRI measurements to demonstrate that hippocampal atrophy even distinguishes subjects with mild age-related memory impairments from control subjects. The researchers also suggested that hippocampal atrophy may predict future cognitive decline.

### MRI Studies of Alcoholic Patients

Like CT studies, MRI analyses can detect cerebral atrophy in chronic alcoholics. The observed changes include increases in the CSF volume as well as decreases in the volume of gray-matter structures (e.g., part of the temporal lobe and of the hippocampus) and white-matter structures (Jernigan et al. 1991; Pfefferbaum et al. 1992; Shear et al. 1994; Sullivan et al. 1995). Some of the observed structural changes may correlate with the subjects’ cognitive functioning as assessed by neuropsychological measures. For example, MRI studies of KS patients revealed more extensive nerve cell loss in the diencephalon (a brain region beneath the cortex that includes the thalamus and hypothalamus) than in alcoholics without the syndrome (Jernigan et al. 1991). Other studies of chronic alcoholics have reported a correlation between impaired neuropsychological functioning and increased CSF volume in the ventricles and sulci, although no consistent relationships have been found to exist between cognitive measures and the volumes of specific cortical or subcortical gray-matter structures (Jernigan et al. 1991). The researchers suggest that this apparent discrepancy may be due, at least in part, to the considerable variability among individuals in the volumes of gray matter structures. Moreover, most neuropsychological tests require the function of multiple brain systems, making correlations between test scores and a single brain structure less likely.

## Functional Imaging

Structural imaging approaches detect changes in brain structure but allow no conclusion as to whether these changes actually affect brain function. In contrast, functional imaging technologies, such as PET and SPECT, permit the visualization of brain functions by measuring variables such as cerebral metabolism of glucose and oxygen or regional cerebral blood flow (RCBF). These techniques are based on the observation that brain regions with increased nerve cell activity show increased blood flow and use more nutrients (e.g., oxygen and glucose) than inactive brain regions. Studies using these techniques usually compare brain functions between patient groups (e.g., AD patients or alcoholics) and normal control subjects. In addition, both PET and SPECT offer the opportunity to study components of the chemical systems involved in signal transmission within the brain (see box).

**New Applications of PET and SPECT**

Both positron emission tomography (PET) and single-photon emission computed tomography (SPECT) allow scientists not only to measure physiological variables, such as blood flow in the brain, but also to study various neurochemical systems. For example, tracers exist that can help analyze the distribution of cellular components, such as the docking molecules (i.e., receptors) on the brain cell surfaces to which the molecules mediating nerve-signal transmission (i.e., neurotransmitters) attach. Tracers are available that allow researchers to examine brain structures using the neurotransmitters dopamine and serotonin.

Neurochemical studies are potentially useful for diagnosing dementia associated with defects in cells that use a specific neurotransmitter system—for example, Parkinson’s disease (PD), a neurodegenerative disorder primarily affecting cells using dopamine. In addition, neurochemical studies enable researchers to examine the relationship between specific neurochemical changes and disease symptoms (e.g., how changes in the dopamine system relate to the motor symptoms [i.e., tremors] that occur in PD patients). Similar studies assess the response of various neurotransmitter systems to pharmacological interventions. To date, these types of studies have not been performed in alcohol research.

Both PET and SPECT use molecules labeled with a radioisotope (i.e., radioactive tracers) to study the rates of blood flow and various metabolic processes in the brain. Tracers used for PET usually are radioactive variants of physiological molecules; for example, glucose molecules containing radioactive fluoride are used to study glucose metabolism, and water or oxygen molecules containing radioactive oxygen atoms are used to study RCBF or oxygen metabolism. Tracer molecules for SPECT usually are nonphysiological radioactive substances. The tracers can be injected into the subject’s bloodstream or, in the case of oxygen metabolism, inhaled. Computers convert the data on the distribution of the radioactive tracers in the subject’s brain into images. (For more information on PET and SPECT, see the article by Doria, pp. 261–265.)

Although compared with SPECT, PET images show greater detail and more accurately quantify the processes studied, clinicians and researchers frequently prefer SPECT because it is less costly, more widely available, less technically challenging, and uses tracers that are routinely available in clinical nuclear medicine departments. However, both PET and SPECT data can be distorted by the atrophy that occurs in normal and pathological aging processes. The shrinkage of brain tissue in a certain brain region artificially lowers the overall metabolic rate in that region because fewer cells are available to metabolize glucose or oxygen while simultaneously the amounts of CSF and white matter are increased. Thus, the images may indicate reduced metabolism even when the metabolic rate in the remaining cells is normal. This limitation has received more attention in PET than in SPECT studies, because PET is more frequently used for quantitative analyses. Several researchers have attempted to develop correction factors to compensate for effects of atrophy when calculating metabolic rates (Chawluk et al. 1987, 1990; Marchal et al. 1992), with contradictory results. Technical advances in PET technology, however, should reduce the influence of atrophy and thus minimize this problem in the future.

### PET and SPECT Studies of Neurodegenerative Diseases and Aging

PET and SPECT both have been used extensively to study AD and other neurodegenerative disorders and to relate the physiological changes to cognitive and clinical findings. The observed patterns of changes in blood flow and brain metabolism allow scientists and clinicians to distinguish between these disorders and the normal aging process.

#### Alzheimer’s Disease

**Figure 1:**
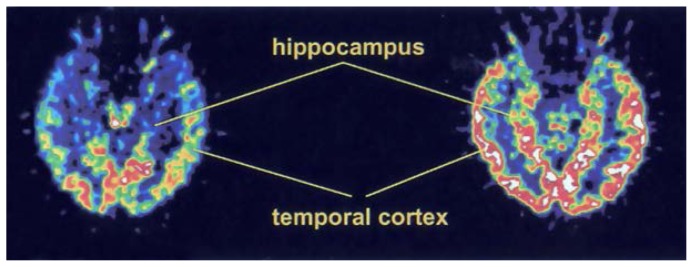
Positron emission tomography images from an Alzheimer’s disease (AD) patient (left) and a control subject (right). These images were taken at a level passing through the middle of the temporal lobe and hippocampus. The AD patient showed reduced metabolism in the temporal cortex and hippocampus.

**Figure 2:**
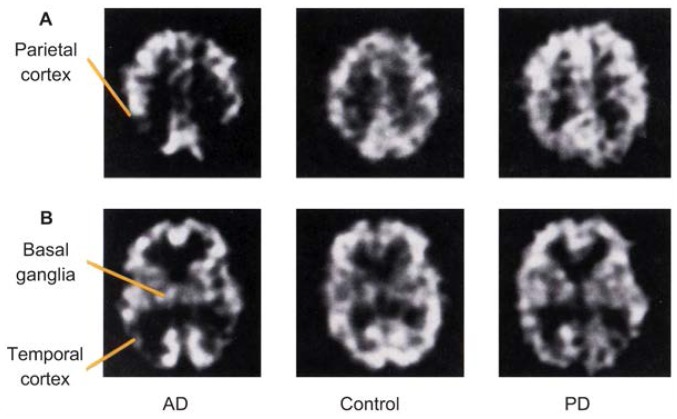
Single-photon emission computed tomography (SPECT) images representing two horizontal cross-sections through the brains of an Alzheimer’s disease (AD) patient, a control subject, and a Parkinson’s disease (PD) patient. The images in row A include the parietal cortex; the images in row B include the basal ganglia and the temporal cortex. Compared with the control subject, the AD patient exhibits strongly reduced blood flow in the parietal and temporal cortices. In the PD patient, blood flow in the pari­etal and temporal cortices is reduced compared with that in the control subject but not as much as in the AD patient.

PET studies consistently have shown characteristic patterns of blood flow and metabolism that are useful for understanding the progression and pathophysiology^4^ of AD as well as the relationship between cognitive deficits and neurophysiological measurements. The predominant finding is a decrease in RCBF, glucose metabolism, and oxygen metabolism in the temporal and parietal lobes, with increasing involvement of other brain regions—especially the frontal lobes—as the disease progresses (figure 1). These localized decreases in blood flow and metabolism correspond to the regional distribution of nerve cell damage in AD (Friedland et al. 1985). Studies examining the relationship between glucose metabolism and neuropathological markers of AD (i.e., neurofibrillary tangles and senile plaques [see sidebar]) in more detail found that glucose metabolism was low in areas with a high density of neurofibrillary tangles but did not correlate with the distribution of senile plaques (DeCarli et al. 1992). Similarly, the presence of neurofibrillary tangles, but not senile plaques, also correlated with dementia severity (Arriagada et al. 1992). SPECT studies using blood flow tracers confirmed the pattern of reduced metabolism in the parietal and temporal lobes observed with PET (figure 2) and established a correlation between the degree of RCBF reduction in these structures and the severity of the patients’ dementia (Jagust et al. 1987).

Several studies found that regional cerebral metabolism and blood flow reductions were associated with declines in specific cognitive functions attributed to those regions. For example, AD patients with predominant visuospatial deficits^5^ had greater metabolic reductions in the right half (i.e., hemisphere) of the brain, whereas patients with predominant language deficits had greater metabolic reductions in the left hemisphere (Foster et al. 1983). Similarly, SPECT studies demonstrated a relationship between the pattern of RCBF changes and specific cognitive deficits (Eberling et al. 1993). Thus, whereas generally reduced metabolism in the parietal and temporal lobes is typical of AD patients, the specific patterns of affected brain regions can differ significantly among patients, contributing to the heterogeneity in the patients’ clinical presentation.

#### Huntington’s Disease and IVD

PET has been reasonably accurate in separating AD patients from patients with other types of dementia (e.g., Huntington’s disease (HD) and IVD) as well as from normal control subjects (table 1). For example, in contrast to the reduced metabolism in the temporal and parietal lobes that is characteristic of AD, PET studies of HD patients mainly detected reduced metabolism in a subcortical structure called the caudate nucleus (Grafton et al. 1992). IVD, by contrast, does not appear to be characterized by any single metabolic pattern. SPECT studies of IVD also found no single characteristic RCBF pattern, but variously detected blood flow deficits in regions of the temporal, parietal, or frontal lobes as well as patchy global deficits (Eberling et al. 1992; Jagust et al. 1987; Komatani et al. 1988). This variability probably is due to the fact that blood flow interruptions in IVD can occur anywhere in the brain: Some patients show signs of infarcts distributed diffusely throughout subcortical structures, whereas others have more localized lesions in the cortex. Moreover, IVD patients frequently also suffer from AD and thus may exhibit changes typical for both disorders.

#### Parkinson’s Disease

PET studies of Parkinson’s disease (PD) patients found that, as with AD, PD patients often show decreased metabolism in the temporal and parietal lobes (Peppard et al. 1992; Eberling et al. 1994), possibly because some PD patients also may have AD or preclinical AD. This is supported by findings that whereas PD patients both with and without dementia show global metabolic reductions, some demented PD patients (i.e., those most likely also to have AD) show more profound metabolic deficits in the parietal and temporal lobes than nondemented PD patients (Peppard et al. 1992). PD patients also show metabolic deficits in areas of the occipital lobe that generally are unaffected in AD (Eberling et al. 1994).

PET studies also found that PD patients exhibited altered metabolism not only in the cortex but also in subcortical structures. These findings included increased blood flow and metabolism in the basal ganglia (Rougemont et al. 1984) as well as reduced metabolism of the neurotransmitter dopamine in the striatum (Leenders et al. 1986), which reflects the dopamine depletion characteristic for this disease (see sidebar).

SPECT studies of PD patients revealed reduced blood flow to the temporal and parietal lobes (figure 2). For example, Pizzolato and colleagues (1988) found decreased parietal RCBF in PD patients with and without dementia, with the degree of RCBF reduction reflecting the extent of mental deterioration. Jagust and colleagues (1992) also reported decreased RCBF in the temporal lobe compared with control subjects. These researchers were able to establish an association of certain RCBF patterns with specific cognitive deficits in the PD patients: Whereas reduced temporal and parietal RCBF was related to the patients’ global cognitive performance, decreased RCBF in the frontal lobe correlated with more specific cognitive deficits compared with control subjects, even in the absence of dementia.

#### Aging

PET studies of normal aging have led to controversial findings concerning age-related changes in cerebral glucose metabolism, with different studies variously reporting global changes, more localized decreases, or no age-related changes at all (Eberling et al. 1995). These conflicting findings may stem partly from differences in subject selection, with some studies selecting only subjects who are optimally healthy (i.e., they do not suffer from diseases typically associated with aging, such as diabetes or heart disease) and others applying less stringent criteria. Eberling and colleagues (1995) studied a group of optimally healthy older subjects who had no cognitive deficits. The study found decreased temporal lobe metabolism, with the greatest metabolic deficits in brain regions that mediate widespread reciprocal connections between the temporal and frontal lobes and structures of the limbic system; these regions probably play a role in multiple cognitive functions, including memories, and frequently show age-related pathological changes. In general, the metabolic patterns in these healthy subjects were similar to those seen in AD patients but with much less extensive deficits. It also is possible that some of the subjects had preclinical AD; a previous study showed that metabolic deficits characteristic of AD may occur prior to the cognitive deficits associated with the disease (Haxby et al. 1986).

### PET and SPECT Studies of Alcoholism

Based on the effectiveness of functional imaging in monitoring the physiological consequences of neurodegenerative diseases and aging, researchers also have begun using these techniques to study the effects of alcohol and other drugs on the brain. Several reports have assessed the effects of cocaine (Holman et al. 1992, 1993), but few PET and SPECT studies have evaluated the neuropathological consequences of chronic alcoholism, alcoholic dementia, or KS.

#### Chronic Alcoholism

Samson and colleagues (1986) used PET to study cerebral glucose metabolism in six chronic alcoholics without cognitive dysfunction. The most characteristic finding in these patients was reduced regional glucose metabolism in the mesial frontal lobe, a region near the middle of the frontal lobe that is connected with structures of the limbic system and diencephalon and may be involved in memory functions. Other studies found similar results, as well as reduced metabolism in the cerebellum (a structure at the base of the brain involved in the control of movement and speech), in alcoholic patients exhibiting some neuropsychological impairments (Gilman et al. 1990). Still other researchers reported diminished metabolic rates throughout the cortex of alcoholic patients (Wik et al. 1988; Sachs et al. 1987). Thus, it appears that alcoholic patients both with and without cognitive impairment may show metabolic deficits in several brain regions, especially in the mesial frontal lobe. Rarely, however, has research examined the relationship between the observed metabolic changes and specific cognitive impairments. For example, Adams and colleagues (1993) correlated reduced glucose metabolism in the mesial frontal lobe with the patients’ performance on tests measuring cognitive functions (e.g., abstract thinking) that have been associated with this region.

#### Korsakoff’s Syndrome

A few studies have examined glucose metabolism and RCBF in KS patients. Using PET, Kessler and colleagues (1984) noted a global reduction of glucose metabolism in these patients. The specific metabolic patterns varied among the patients: While some had particularly low metabolism in the frontal lobe but elevated metabolism in subcortical structures (e.g., the basal ganglia and the thalamus), others showed the opposite pattern. The interpretation of these findings was limited, however, because the study did not include alcoholics without KS for comparison. Other researchers found that KS patients had reduced glucose metabolism in the frontal and parietal lobes and in the cingulate gyrus (i.e., a part of the limbic system that, among other functions, has been associated with performance on some memory tasks) compared with alcoholic control subjects (Paller et al. 1993).

The reduced metabolism and, thus, the reduced nerve cell activity in the cortex could have two causes. First, it could be the direct result of alcohol-induced damage to nerve cells in the cortex. Alternatively, it could indirectly result from damage to cells in the diencephalon, which are extensively connected with cells in the cortex. Both diencephalic and cortical damage and dysfunction have been observed in KS patients, suggesting that the memory deficits of KS patients, which often have been attributed to damage to diencephalic structures, also may be related to functional abnormalities in the cortex. For example, a SPECT study of RCBF in KS patients found a correlation between impaired performance on memory and orientation tests and diminished RCBF in the frontal lobes (Hunter et al. 1989).

## Conclusions

Imaging technologies, especially new functional imaging methods, have significantly enhanced our understanding of the pathophysiological processes in the brain that lead to or result from various types of neurodegenerative diseases and other causes of dementia as well as the normal aging process. These studies have helped define different types of dementia (e.g., AD versus IVD) and predict their clinical course. Based on imaging studies, we now know, for example, that the size of the hippocampus can predict subsequent cognitive decline in aging subjects (deLeon et al. 1993) and that the extent of RCBF reduction in the temporal lobe indicates the rate of cognitive decline in AD patients (Wolfe et al. 1995). Other studies have identified people at risk for AD based on reduced glucose metabolism in the parietal cortex, and long-term studies of these people may allow researchers to monitor treatment responses in the earliest stages of the disease (Small et al. 1995).

Functional imaging technologies such as SPECT and PET also potentially are useful for investigating some of the causes and consequences of alcoholism. For example, imaging studies may help to disentangle the effects of aging from the effects of alcoholism. Studies demonstrating similar changes in brain metabolism among older people and in alcoholics indicate that chronic alcohol consumption may induce or accelerate aging processes in the brain. This hypothesis is supported by imaging analyses showing that the volume of the hippocampus decreases both in chronic alcoholics and in the course of aging; this effect is more pronounced in older alcoholics than in younger alcoholics (Sullivan et al. 1995).

Imaging studies also may help researchers relate physiological changes in alcoholics to observed cognitive deficits. In particular, the development of new SPECT and PET radiotracers should permit the evaluation of a number of neurotransmitter systems and the relationship between physiological changes in these systems and specific cognitive deficits. Imaging studies also may be useful for defining specific alcohol-related neurobehavioral syndromes (e.g., chronic alcoholism without dementia, alcoholic dementia, and KS) in the same way they have helped define different types of dementia. Finally, imaging might be used to identify alcoholics likely to develop dementia as well as to predict the progression of cognitive changes. To realize their potential and to determine the relationships between functional changes, clinical presentation, and specific cognitive impairments, however, future imaging studies must clearly characterize the neurobehavioral status of their subjects and include appropriate control groups.

## Figures and Tables

**Table 1:** Comparison of the Changes in Brain Metabolism Associated With Neurodegenerative Diseases, Alcoholism, and Aging.

Condition	Affected Brain Regions
Alzheimer’s disease	Reduced metabolism in the temporal lobes and parietal lobes
Huntington’s disease	Reduced metabolism in the caudate nucleus
Parkinson’s disease	Reduced metabolism in the temporal and parietal lobes, particularly in patients with cognitive decline Increased blood flow and metabolism in the basal ganglia (observed only in some studies)
Normal aging	Reduced metabolism in the temporal lobe but to a lesser extent than in Alzheimer’s disease
Chronic alcoholism	Reduced metabolism in several brain regions, particularly in the mesial frontal lobe
Korsakoff’s syndrome	Reduced metabolism in the frontal, parietal, and cingulate lobes

## Footnotes

1 For a definition of this and other technical terms see the central glossary, pp. 293–295.

2 The gray matter of the brain mainly is composed of the bodies of the nerve cells, whereas the white matter primarily is made up of the cells’ long extensions, or axons.

3 For the location of this and other brain structures, see the figures on pp. 293–295.

4 Pathophysiology is the study of the processes in the body that lead to the signs and symptoms of a disease.

5 Visuospatial functions include reading maps, solving jigsaw puzzles, and performing motor skills involving spatial perception.
